# Laparoscopic hepatectomy for elderly patients: Major findings based on a systematic review and meta-analysis

**DOI:** 10.1097/MD.0000000000011703

**Published:** 2018-07-27

**Authors:** Ke Chen, Yu Pan, Hendi Maher, Bin Zhang, Xue-yong Zheng

**Affiliations:** a Department of General Surgery, Sir Run Run Shaw Hospital, School of Medicine, Zhejiang University, Zhejiang Province, China; b School of Medicine, Zhejiang University, Zhejiang Province, China.

**Keywords:** elderly, hepatectomy, laparoscopy, liver neoplasm, meta-analysis

## Abstract

**Background:**

As the general population continues to age, there is an increase need for surgical management of elderly patients. Compared to open hepatectomy (OH), laparoscopic hepatectomy (LH) offers earlier mobilization, less blood loss, and shorter postoperative hospital stay. However, whether these advantages of LH over OH are retained in elderly patients remains to be clarified. Therefore, in this study, we sought to evaluate the feasibility, safety, and potential benefits of LH for elderly patients.

**Methods:**

A systematic search of PubMed, Embase, Cochrane Library, and Web of Science was performed to identify studies that compared LH and OH. Studies comparing LH in elderly and LH in nonelderly patients were also identified. Outcomes of interest included conversion rate, operative time, intraoperative estimated blood loss, length of hospital stay, rate and type of morbidity, mortality rate, margin status (R0), and long-term oncologic outcomes.

**Results:**

Nine studies met our inclusion criteria for this analysis. Of these, 5 compared LH and OH in elderly patients, 3 compared LH in elderly and nonelderly patients, and 1 included both outcomes. Compared to those with OH, elderly patients who underwent LH had similar operative times [weighted mean difference (WMD) = 1.15 minutes; 95% confidence interval (CI): −28.28–30.59, *P* = .94], less intraoperative blood loss (WMD = −0.71 mL; 95% CI: −1.29 to −0.16, *P* = .01), a lower rate of transfusion [risk ratio (RR) = 0.61, 95% CI: 0.40–0.94, *P* = .02], comparable R0 rates (RR = 1.01; 95% CI: 0.96–1.07, *P* = .70), less postoperative complications (RR = 0.61, 95% CI: 0.48–0.76, *P* < .01), and shorter hospital stay (WMD = −3.22 days; 95% CI: −4.21 to −2.23, *P* < .01). The limited long-term outcomes indicated that survival status was comparable between LH and OH for elderly patients. The pooled outcomes for elderly versus nonelderly patients indicated that the safety and effectiveness of LH over OH in elderly patients was not inferior to those in nonelderly patients.

**Conclusion:**

Our results indicate that LH is a feasible and safe alternative to OH in elderly patients, providing a lower rate of morbidity and favorable postoperative recovery and outcomes.

## 1 Introduction

Since laparoscopic hepatectomy (LH) was first reported in 1996,^[1]^ the technique has been rapidly adopted around the world. As a minimally invasive technique, LH provides several advantages, over an open approach [open hepatectomy (OH)], including decreased pain, better cosmesis, faster recovery, lesser complications, and earlier ambulation compared to open surgery.^[2–5]^ In 2014, a consensus conference on LH held in Morioka supported LH as a safe and effective approach for the management of liver disease.^[6]^ In some high-volume academic centers, the use of laparoscpic techniques has been extended to major hepatectomy and some resections of special liver segments.^[7–10]^ However, the application of LH in special clinical populations, such as patients with cirrhosis and other severe comorbidities,^[11,12]^ or elderly patients remains to be clarified.

With life expectancy continuing to increase, the number of elderly individuals is constantly growing, worldwide.^[13]^ The number of elderly patients with pre-existing diseases, such as cardiovascular disease and diabetes mellitus, is also increasing significantly. As such, the minimally invasive nature of laparoscopic surgery would be beneficial for the treatment of elderly individuals. However, the research specifically studying the application of LH in the elderly population is still limited and, therefore, the true merits of LH for the surgical management of elderly patients are still uncertain. Accordingly, our aim in this study was to comprehensively collect relevant evidence and conduct a systematic review and meta-analysis to assess the feasibility, safety, and potential benefits of the use of LH for the surgical management of elderly patients.

## 2 Materials and methods

### 2.1 Systematic literature search

Studies were identified by searching electronic databases and by scanning the reference lists of articles. Systematic searches of PubMed, Embase, Cochrane Library, and Web of Science were performed to identify articles published up to September 2017, using the following search terms, either independently or in combination: “laparoscopy,” “laparoscopic,” “minimally invasive,” “hepatectomy,” “liver resection,” “hepatic resection,” “liver cancer,” “liver neoplasm,” “liver tumor,” “hepatic cancer,” “hepatic neoplasm,” “hepatic tumor,” “elderly,” “geriatric,” “old,” and “aged.” All eligible studies published in English were retrieved; the reference list of retrieved studies was manually searched to identify further potentially relevant publications.

### 2.2 Eligibility criteria

The inclusion criteria for systematic review and meta-analysis were prospective or retrospective case series studies assessing surgical outcomes of LH for elderly and nonelderly patients with definite age cutoff points, or comparative studies of LH and OH for elderly patients. The following studies or data were excluded: case reports, reviews, letters, editorials, and studies without a control group and inclusion of patients who underwent major digestive surgery, other than LH. In cases of overlap between authors or centers among different studies, the higher-quality and/or more recent study was selected. Of note, studies from the same authors or centers but with different patient cohorts were included.

### 2.3 Data extraction and quality assessment

Two investigators (KC and YP) independently assessed publications for inclusion in the article. Discrepancies between the 2 reviewers were resolved via discussions with the third senior author (XYZ). Data extracted from eligible studies included the baseline characteristics, conversion rate, operation time, estimated blood loss, length of hospital stay, morbidity, mortality, margin distance, and long-term oncologic outcomes. The postoperative morbidity was cataloged according to the Clavien-Dindo Classification. Minor complication refers to grades I and II complications, and major complication include grades III to V complications. The Newcastle-Ottawa Quality Assessment Scale (NOS) was used to evaluate the quality of the research included. The scale ranges from 0 to 9 stars: research with a score ≥6 could be deemed methodologically sound.

### 2.4 Statistical analysis

Dichotomous variables, such as postoperative morbidities, between surgical methods, were compared using the risk ratio (RR), with 95% confidence interval (CI). Continuous parameters, such as operative time and volume of blood loss, were compared using a weighted mean difference (WMD), with 95% CI. The means and standard deviations (SDs) were estimated those as described by Hozo et al,^[14]^ if the research offered medians and ranges rather than means and SDs. Statistical heterogeneity, which indicated between-study variance, was evaluated according to the Higgins *I*^2^ statistic.^[15]^ Heterogeneity was evaluated by Cochran *Q* statistic and *I*^2^. If data were not significantly heterogeneous (*P* > .05 or *I*^2^ < 50%), the pooled effects were calculated using a fixed model. Otherwise, the pooled effects were calculated using a random-effects model. According to the general complication, the bias of potential publication was determined by carrying out informal visual inspection of funnel plots. All statistical tests were performed using Review Manager (version 5.1; The Cochrane Collaboration, Oxford, England).

### 2.5 Ethics statement

This study was a secondary analysis regarding human subject data published in the public domain; thus, no ethical approval was required.

## 3 Results

### 3.1 Study eligibility

**Figure 1:**
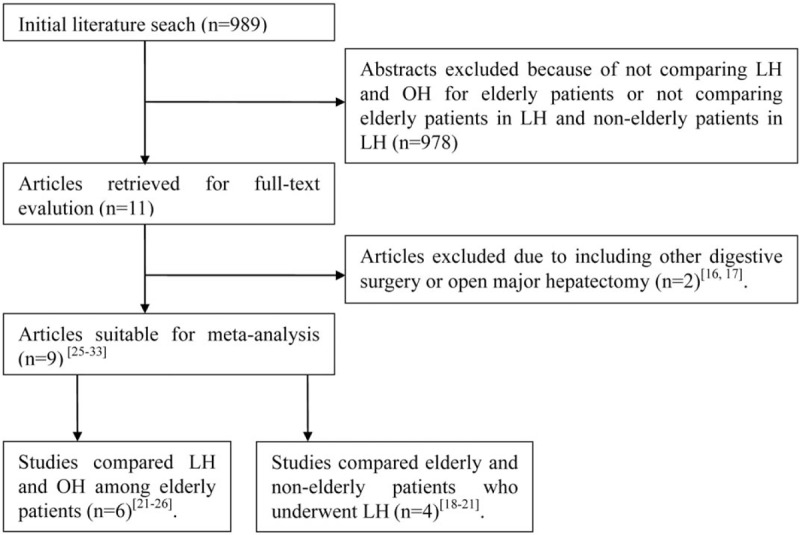
Flow chart of literature search strategies. LH = laparoscopic hepatectomy.

Our search identified 989 articles; of these, 978 were excluded based on screening of the title and abstract. Two further articles were excluded after full-text review due to their inclusion of other digestive surgeries or open major hepatectomy.^[16,17]^ Finally, 9 studies were selected for further meta-analysis.^[18–26]^ Of these, 5 studies compared LH and OH among elderly patients,^[22–26]^ 3 evaluated the safety and feasibility of LH among elderly patients compared to nonelderly patients also receiving LH^[18–20]^ and 1 compared the outcomes of LH for both elderly patients with nonelderly patients to LH and OH among elderly patients.^[21]^ No randomized controlled trial (RCT) was found. A flow chart of the search strategies, including reasons for exclusion of studies, is shown in Figure 1.

### 3.2 Study characteristics

A total of 830 patients were included in the study analysis with 388 undergoing LH (46.8%), and 442 undergoing OH (53.2%). As for the analysis of elderly versus nonelderly patients in LH, 112 elderly patients received LH (28.9%), and 276 geriatrics underwent LH (71.1%). The characteristics of the included studies are summarized in Tables 1 and 2. They represent international populations (2 France, 2 Italy, 1 Japan, 1 Chinese Hong Kong, 2 Chinese Mainland, and 1 European multicenter). The majority of studies report the experience at a single center, whereas 2 studies conducted research over multiple institutions.^[21,26]^ Five studies were case-matched research studies.^[19,22–24,26]^ Five studies used 70 years as the age cutoff the elderly label,^[18,19,22,23,26]^ whereas 1 study used 65 years^[24]^ and the other 3 studies used 75.^[20,21,25]^ The indications of 2 studies included benign liver lesions and malignancy.^[20,21]^ The indications of the remaining 7 studies were reported as malignant, with colorectal metastases the most frequently recorded. One study was only restricted to primary hepatocellular carcinoma.^[25]^ The majority type of intervention was total or pure laparoscopic hepatic resection, but 1 study used hand-assisted or hybrid procedures.^[20]^ All of the included studies graded morbidity according to the Clavien-Dindo Classification. The definition of mortality was 90 days in 5 studies, which included perioperative death cases,^[18,19,21,25,26]^ and 1 study used 30-day mortality.^[24]^ Four studies reported mid- or long-term postoperative survival results.^[18,19,24,26]^ The quality of the research included was generally moderate or satisfactory. NOS shows that 2 out of the 9 studies observed had 6 stars, 3 had 7 stars, and 4 got 9 stars. Table 3 shows the evaluation of quality according to the NOS.

**Table 1:**
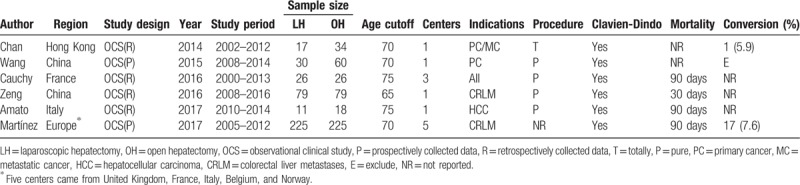
Summary of studies included in the meta-analysis of laparoscopic hepatectomy versus open hepatectomy among elderly patients.

**Table 2:**
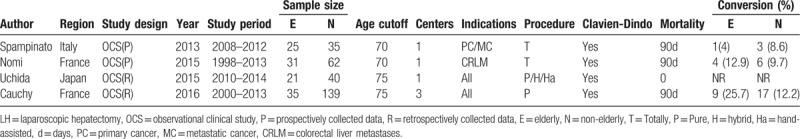
Summary of studies included in the meta-analysis of elderly versus nonelderly patients who underwent laparoscopic hepatectomy.

**Table 3:**
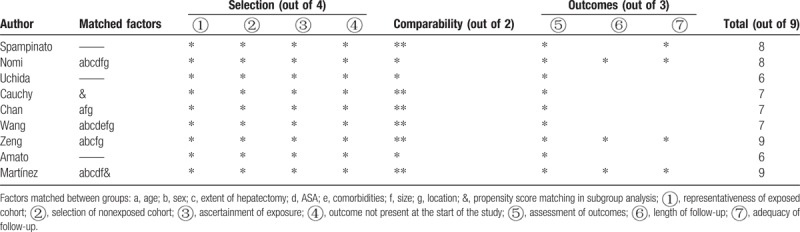
Quality assessment based on the Newcastle-Ottawa Quality Assessment Scale for observational studies.

### 3.3 Short-term outcomes of LH versus OH among elderly patients

**Figure 2:**
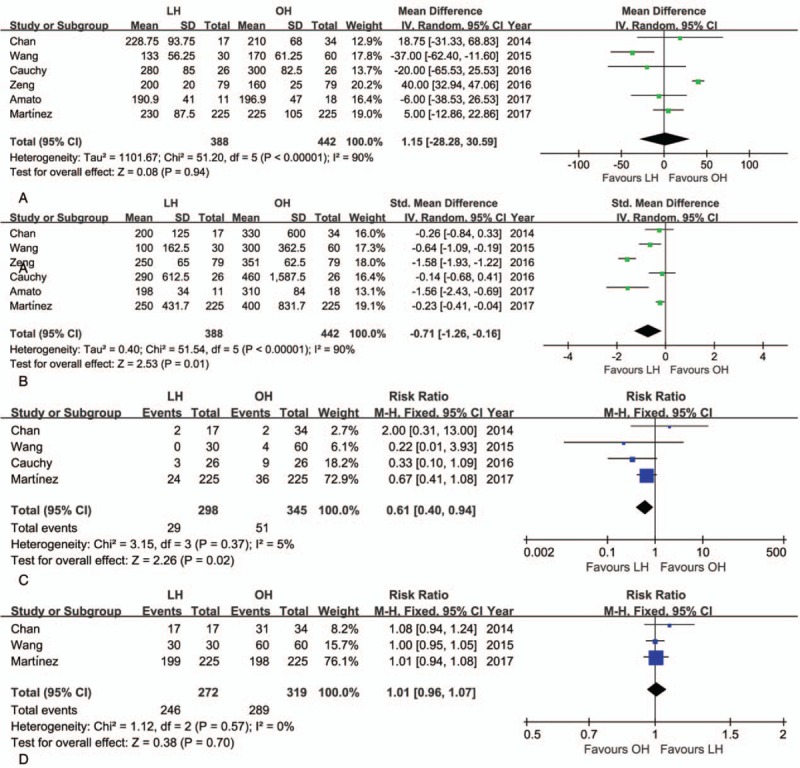
Forest plot of the meta-analysis for short-term outcomes of LH versus OH among elderly patients (intraoperative effect). A, Operative time. B, Blood loss. C, Transfusion. D, R0 rate. CI = confidence interval, LH = laparoscopic hepatectomy, OH = open hepatectomy.

**Figure 3:**
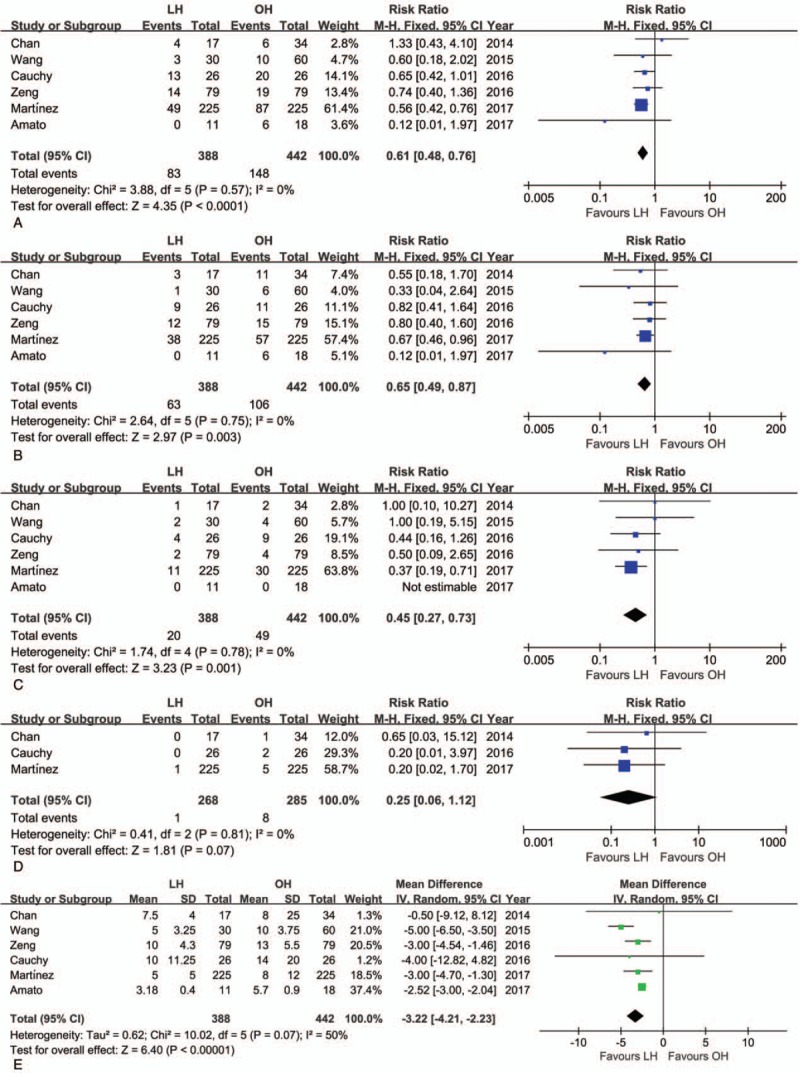
Forest plot of the meta-analysis for short-term outcomes of LH versus OH among elderly patients (postoperative recovery). A, Overall morbidity. B, Minor complications. C, Major complications. D, Mortality. E, Hospital stay. CI = confidence interval, LH = laparoscopic hepatectomy, OH = open hepatectomy, SD = standard deviation.

The mean operative time was similar for LH and OH (WMD = 1.15 minutes; 95% CI: −28.28–30.59, *P* = .94; Fig. 2A). However, the volume of intraoperative blood loss was lower for LH than OH (WMD = −0.71 mL; 95% CI: −1.29 to −0.16, *P* = .01; Fig. 2B), as was the need for transfusion (RR = 0.61, 95% CI: 0.40–0.94, *P* = .02; Fig. 2C). The rate of R0 resection was not significantly different between LH and OH (RR = 1.01; 95% CI: 0.96–1.07, *P* = .70; Fig. 2D). Postoperative morbidity was lower for LH than OH (RR = 0.61, 95% CI: 0.48–0.76, *P* < .01; Fig. 3A). Specifically, both the rate of minor and major postoperative complications was lower for LH than for OH (minor complications, RR = 0.65, 95% CI: 0.49–0.87, *P* < .01; Fig. 3B; major complications, RR = 0.45, 95% CI: 0.27–0.73, *P* < .01; Fig. 3C). Pooled data analysis identified a trend toward a lower rate of mortality for LH than OH (RR = 0.25, 95% CI: 0.06–1.12, *P* = .07; Fig. 3D). The postoperative complications reported in the included studies are summarized in Table 4.

**Table 4:**
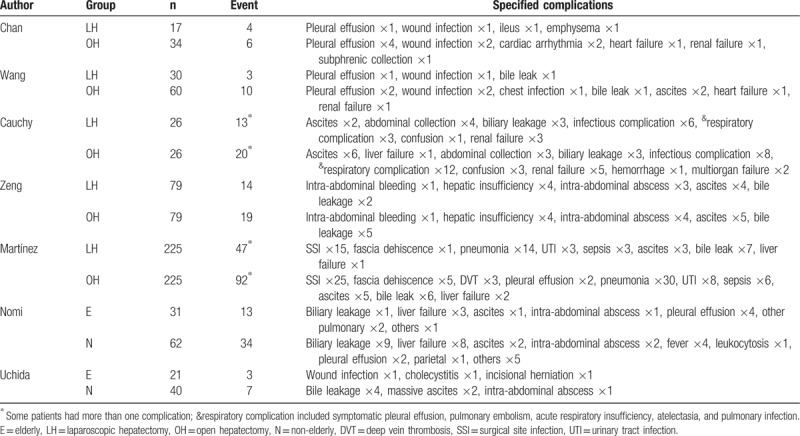
Systematic review of postoperative complications.

Hospital stay was shorter for LH than OH (WMD = −3.22 days; 95% CI: −4.21 to −2.23, *P* < .01; Fig. 3E). The short-term outcomes of LH and OH among elderly patients are summarized in Table 5. Postoperative hepatic function was evaluated in 2 studies.^[22,23]^ Wang et al^[23]^ reported the same trends in postoperative levels of alanine aminotransferase (ALT), aspartate aminotransferase (AST), and total bilirubin (TB), all of which peaked on postoperative day 1, with levels normalizing in postoperative day 3. However, median serum ALT and AST levels were significantly lower in the LH group than OH group during the first 3 days after operation.^[23]^ On the contrary, Chan et al^[22]^ also reported a comparable postoperative trend, but with no difference in ALT or TB between the 2 groups at each time point of measurement after surgery.

**Table 5:**
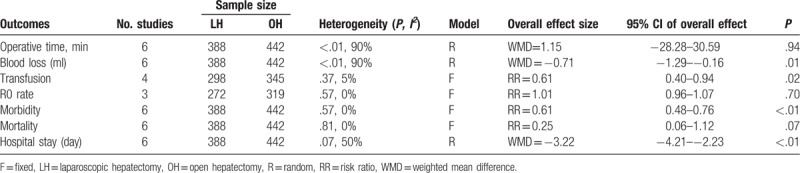
Pooled outcomes of meta-analysis of laparoscopic hepatectomy versus open hepatectomy among elderly patients.

### 3.4 Short-term outcomes of elderly versus nonelderly patients who underwent LH

**Figure 4:**
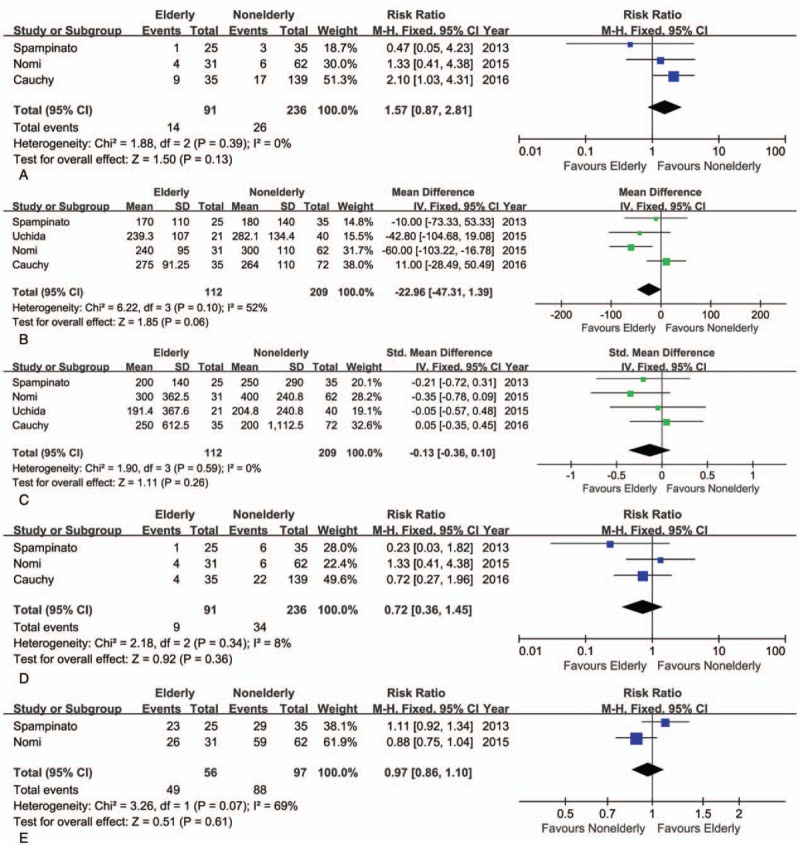
Forest plot of the meta-analysis for short-term outcomes of elderly versus nonelderly patients who underwent laparoscopic hepatectomy (LH; intraoperative effect). A, Conversion. B, Operative time. C, Blood loss. D, Transfusion. E, R0 rate. CI = confidence interval, SD = standard deviation.

**Figure 5:**
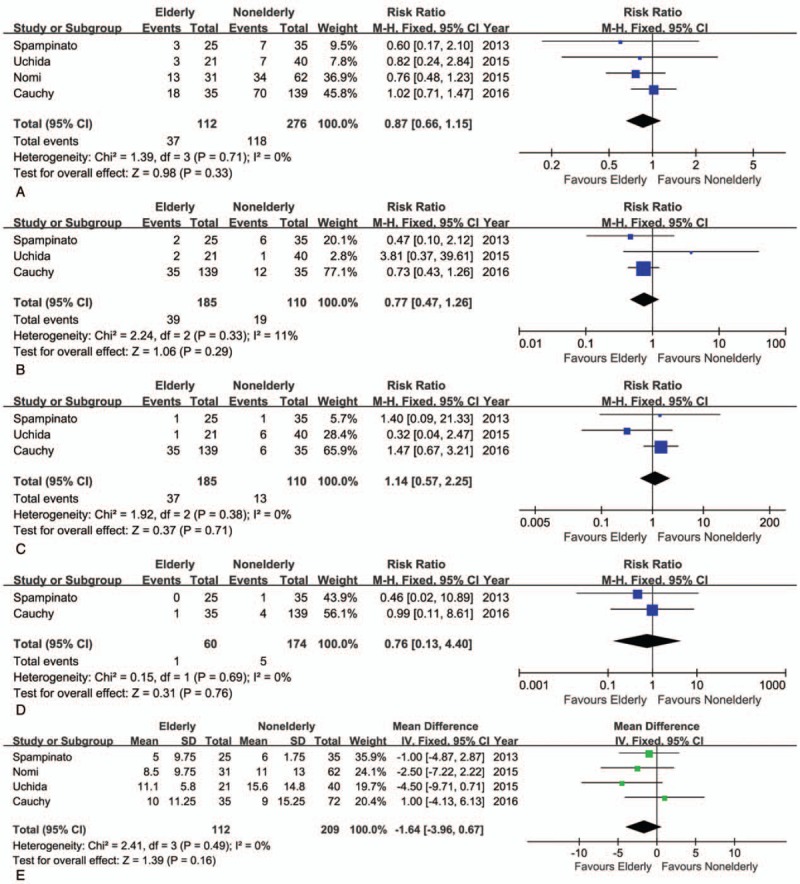
Forest plot of the meta-analysis for short-term outcomes of elderly versus nonelderly patients who underwent laparoscopic hepatectomy (LH; postoperative recovery). A, Overall morbidity. B, Minor complications. C, Major complications. D, Mortality. E, Hospital stay. CI = confidence interval, SD = standard deviation.

The conversion rate from LH to OH was similar between elderly and nonelderly patients (RR = 1.57, 95% CI: 0.87–2.81, *P* = .13; Fig. 4A). Although the mean operative time was shorter in the elderly than nonelderly group, this difference was not statistically significant (WMD = −22.96 minutes; 95% CI: −47.31–1.39, *P* = .06; Fig. 4B). The intraoperative volume of blood loss was similar between groups (WMD = −0.13 mL; 95% CI: −0.36–0.10, *P* = .26; Fig. 4C), as was the transfusion rate (RR = 0.72, 95% CI: 0.36–1.45, *P* = .36; Fig. 4D). Moreover, on meta-analysis, no significant difference between elderly and nonelderly patients was identified with regard to the R0 rate (RR = 0.97, 95% CI: 0.86–1.10, *P* = .61; Fig. 4E). There was neither significant difference in the rate of overall postoperative morbidity between the elderly and nonelderly groups (RR = 0.87, 95% CI: 0.66–1.15, *P* = 0.33; Fig. 5A), nor in the rate of minor or major complications (minor: RR = 0.77, 95% CI: 0.47–1.26, *P* = .29; major: RR = 1.14, 95% CI: 0.57–2.25, *P* = .71; Fig. 5B, C). As well, the mortality rate was also comparable (RR = 0.76, 95% CI: 0.13–4.40, *P* = .76) (Fig. 5D). The postoperative complications reported in the included studies are summarized in Table 4. On pooled data analysis, there was no significant difference in the length of hospital stay between the elderly and nonelderly groups (WMD = −1.46 days, 95% CI: −3.96–0.67, *P* = .16; Fig. 5E). The short-term outcomes for the elderly and nonelderly groups are summarized in Table 6.

**Table 6:**
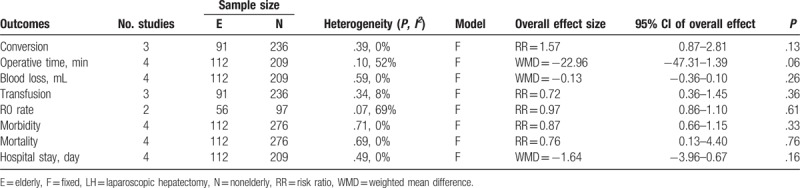
Pooled outcomes of meta-analysis of elderly versus nonelderly patients who underwent laparoscopic hepatectomy.

### 3.5 Long-term outcomes

Follow-up time, recurrence rate, and long-term survival are summarized in Table 7. The long-term survival rates of elderly patients were reported in 2 studies, with no considerable difference in the survival rates between LH and OH identified.^[24,26]^ A meta-analysis of survival rate could not be performed due to the limited dataset available. Long-term outcomes between elderly and nonelderly patients who underwent LH was reported in 2 studies,^[18,19]^ with no significant between-group difference identified (RR = 0.78, 95% CI: 0.56–1.09, *P* = .14).

**Table 7:**
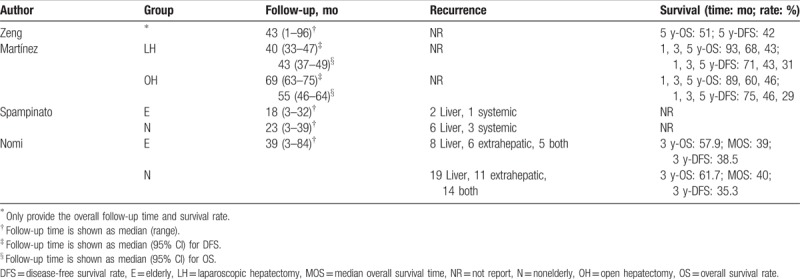
Summary of recurrence and long-term survivals.

### 3.6 Publication bias

**Figure 6:**
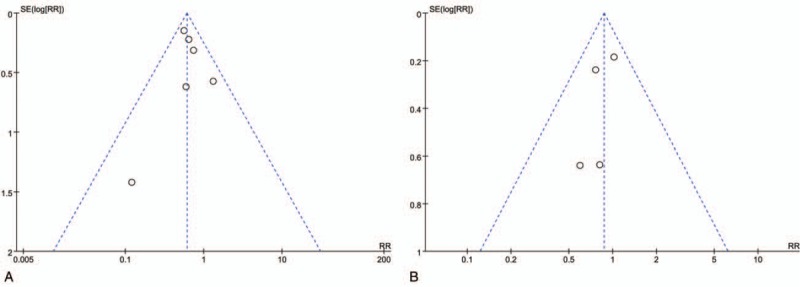
Funnel plots of the overall postoperative complications rates. A, laparoscopic hepatectomy (LH) versus open hepatectomy (OH) among elderly patients. B, Elderly versus nonelderly patients who underwent LH.

The funnel plot for studies reporting the RRs of postoperative morbidity was used to detect publication bias. The plots standing for the studies distributed symmetrically. This result suggests that the publication bias is acceptable (Fig. 6).

## 4 Discussion

With increasing life expectancy, there is an increasing number of elderly patients being referred for surgical hepatic resection,^[13]^ although these patients do present with lower functional reserve of the liver and increased comorbidities compared to younger patients. Considering that the rates of morbidity and mortality after LH are similar to those for OH and the additional benefits of LH, including minimal invasiveness, more rapid recovery, and less pain,^[2–4,27–29]^ LH could be of benefit for elderly patients. Several previous studies have demonstrated that minimally invasive techniques provided favorable outcomes for the surgical management of benign and malignant diseases in elderly patients.^[30–32]^ However, there are limited data about the efficacy of LH in the elderly, which compelled us to conduct this meta-analysis.

Current evidence supports LH as a safe and practical alternative to OH for liver resection in elderly patients, providing several advantages over OH in this clinical population, including lower volume of intraoperative blood loss, shorter hospital stay, and lower postoperative morbidity. Of importance, the rate of postoperative morbidity and mortality after LH was not different between elderly and nonelderly patients. In general, for patients older than 70 years, a rate of morbidity of 20% and of mortality of 6% has been reported during hospitalization, with these rates increasing as a function of age.^[33]^ One multicenter study performed a subgroup analysis by dividing the total cohort into 3 subgroups based on age (70–74, 75–79, and >80 years),^[34]^ showing that the advantages of LH, including lower volume of blood loss, lower overall rate of postoperative morbidity, and shorter hospital stay, were evident in the 70 to 74 years subgroup, with a gradual loss of these advantages with increased age.^[13]^

In our meta-analysis, the overall rate of postoperative complication was 21.4% (83/388 cases) for LH and 33.5% (148/442 cases) for OH, this difference being significant. Moreover, LH was associated with a lower risk of both minor (grade I–II, RR = 0.65) and major (grade III–V, RR = 0.45) complications. In certain situations, minor complications can evolve into major complications or even death, especially in patients with a poor physiological status. Although there was no statistical difference in the rate of postoperative mortality between LH and OH, we did identify a trend favoring LH, which we attributed to the minimally invasive nature of LH. Specifically, the smaller surgical incision required for LH would reduce exposure to bacteria and, subsequently, decreases incisional complications. This milder surgical trauma decreases the acute phase reaction. Moreover, accurate vascular control further reducing the volume of intraoperative blood loss with less disruption of homeostatic regulation. As for OH, the Pringle maneuver and intravenous fluid restriction can also be used for LH, as required,^[21,23,25]^ which is especially effective in controlling bleeding and providing a clear surgical view under laparoscopy. The decrease in blood loss with LH, compared to OH, results in a steady hemodynamic alteration and, thus, a lower rate of transfusion for LH than OH (RR = 0.61). This lower rate of blood transfusion with LH is clinically important, considering the risks associated with blood transfusion, including immune modulation, systemic infection, and transmission of certain diseases, with these risks increasing as a function of the units of blood transfused.

Pneumoperitoneum with LH is a concern for surgeons, being associated with a higher rate of gas embolism rate and increased risk for adverse respiratory and cardiovascular events. Abundant hepatic sinusoid and capillaries are unavoidably exposed to gas during liver resection. However, we did not identify a difference in the rate of cardiopulmonary complication between LH and OH; in fact, LH was associated with a lower risk of pulmonary complication. Similarly, using a multi-institutional propensity score analysis, Fuks et al^[28]^ reported a lower risk of pulmonary complications for LH than OH among patients undergoing major liver resection.

Laparoscopic surgery is considered to be a more complex and time-consuming approach than the conventional open approach. Specifically, elaborate manipulation, hazy surgical vision caused by smog during resection, and unexpected bleeding make laparoscopic liver resection more time consuming and technically demanding than OH. However, with advances in surgical instruments and accumulation of technical expertise, the LH procedure has been facilitated using practical solutions, such as the introduction of a special aspiration system, prepared Pringle maneuver, and intravenous fluid restriction. Notably, in our center, we conventionally use the Laparoscopic Peng Multifunction Operative Dissector (LPMOD), a special instrument that combines the electrotome with an aspirator.^[35]^ As such, using the LPMOD, blunt dissection and aspiration can be performed alternatively by 1 surgeon, making the surgical procedure more fluid, such that LH can be performed in the same timeframe, or even faster, than OH. In our body research evidence, there was no difference in operative time between LH and OH. Moreover, LH does not require a long incision, as for OH.

Achieving a tumor-free surgical margin is of great importance for malignant liver disease. Three pooled studies in our meta-analysis reported on the status of surgical margin, including 1 study that focused on metastatic cancer in the liver, 1 on primary liver cancer and the last on both metastatic and primary liver malignancy. These 3 studies reported achievement of an R0 margin with LH, with the rate of R0 margin with LH not being inferior to OH. The precise localization of the intraoperative tumor is critical for LH, as direct tactile assessment of the liver is not possible. To overcome this limitation, the use of intraoperative ultrasonography is recommended to enhance tumor detection.^[23,25]^ Two included studies did not identify a significant difference in survival rate between LH and OH performed in elderly patients.^[24,34]^ Therefore, the disease itself influences oncological outcomes, rather than the surgical approach or a patient's age. However, because specific data for elderly patients are still lacking, studies with a large sample size and long-term follow-up to confirm our findings are needed.

Our research has some limitations, as follows. First, in the absence of RCTs examining LH and OH among elderly patients, a selection bias regarding the selection of surgical management used is unavoidable.^[36]^ However, it is important to note that approximately 80% of patients were matched using a propensity score that corresponds to the method among studies included in our analysis. Second, studies did not evaluate preoperative risk and, therefore, it is unknown if the type of surgical management for elderly patients was based, in part, on age itself. A preoperative risk evaluation, using geriatric scores appropriate for an elderly population, is necessary and should include collaboration among geriatricians, anesthesiologists, oncologists, and surgeons. However, none of the included studies evaluated such information.^[37–41]^ As well, there was insufficient data at the extreme age, specifically patients above the age of 80 years who are clearly at a higher risk of death. There are well-known differences in fitness and physical health among individuals 70 and 80 years old, with the risk of death increasing as a function of age. However, only 1 study included in our analysis specifically evaluated octogenarian patients to assess age-specific differences in outcomes of LH and OH, which concluded that the advantages of LH may be less evident with increased age and may disappear in octogenarians. Therefore, whether LH is as good for extremely old patients as young and elderly patients requires further research for confirmation.

## 5 Conclusion

According to our data, laparoscopic liver resection is a safe and effective technique for elderly individuals. With regard to short-term outcomes, LH provides several benefits over OH for elderly patients, including less intraoperative blood loss, lower postoperative morbidity, and earlier recovery. We believe that age is not a contraindication for either conventional or laparoscopic approach.

## Author contributions

**Data curation:** Yu Pan.

**Formal analysis:** Ke Chen.

**Methodology:** Xue-yong Zheng.

**Supervision:** Xue-yong Zheng.

**Writing – original draft:** Ke Chen, Hendi Maher.

**Writing – review and editing:** Bin Zhang, Xue-yong Zheng.
